# Retrospective analysis of efficacy and safety of oral paclitaxel for treatment of various cancers in dogs (2017–2021)

**DOI:** 10.1002/vms3.829

**Published:** 2022-05-27

**Authors:** Hyung‐Kyu Chae, Ye‐In Oh, Sumin Park, Ju‐Hyun An, Kyoungwon Seo, Kyuyong Kang, Seung‐nam Chu, Hwa‐Young Youn

**Affiliations:** ^1^ Laboratory of Veterinary Internal Medicine, College of Veterinary Medicine Seoul National University Seoul Republic of Korea; ^2^ College of Veterinary Medicine and the Research Institute for Veterinary Science Seoul National University Seoul Republic of Korea; ^3^ DaeHwa Pharmaceutical Co. Ltd. Pangyo Research Laboratory Sungnam Republic of Korea

**Keywords:** cancer, chemotherapy, dog, efficacy, paclitaxel, safety

## Abstract

**Background:**

In humans, several safety evaluations have shown minimal adverse events with oral paclitaxel; however, its therapeutic efficacy and safety has not been well established in dogs with various cancers.

**Objectives:**

We aimed to retrospectively evaluate the efficacy and safety of oral paclitaxel in dogs with various cancers.

**Methods:**

Twenty‐one dogs diagnosed with various cancers were administered several doses of oral paclitaxel three times a month (group 1) or six times a month (group 2).

**Results:**

The overall response rate was 6.25% (6.25%, complete response; 56.25%, stable disease; 37.5%, progressive disease) in dogs for which the treatment response could be evaluated. The median overall survival (OS) and progression‐free survival (PFS) were 74 and 60.5 days, respectively. Regardless of the administration group, differences in OS and PFS of the two groups did not reach statistical significance. Most dogs tolerated the treatment regimen well, and although minor adverse events were observed in some dogs, they recovered after temporary drug discontinuation, dose reduction or symptomatic treatment. There was no significant difference in the prevalence of adverse events between the two groups.

**Conclusions:**

Based on the observed responses in certain types of cancers and the minimal adverse events, the study findings supported the efficacy and safety of oral paclitaxel administration in dogs. Thus, oral paclitaxel could play a role in the management of cancer in dogs.

## Graphical abstract

The results of this study suggest that oral paclitaxel has the potential to be used as a safe and effective treatment for dogs with various cancers.

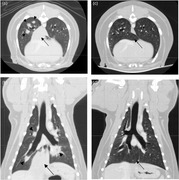

## 1 INTRODUCTION

Paclitaxel was discovered as a part of the National Cancer Institute screening for natural substances with anti‐cancer activity (Cragg, 1998; Khanna et al., 2015). The primary mechanism of paclitaxel is suppression of microtubule spindle dynamics by targeting tubulin, resulting in inhibition of mitosis and induction of cell death in cancer (Manfredi & Horwitz, 1984; Zhang et al., 2014). It is also known to have anti‐angiogenic effects through downregulation of vascular endothelial growth factor (Bocci et al., 2013). In human medicine, paclitaxel has been used alone and in combination with other drugs to treat various cancers, such as advanced ovarian cancer, metastatic breast cancer, non‐small cell lung cancer, bladder cancer, and head and neck cancer (Hajek et al., 1996). In previous studies evaluating the safety of paclitaxel for various cancers, common adverse events included fatal hypersensitivity reactions, nausea, vomiting, loss of appetite and myelosuppression (neutropenia and thrombocytopenia) (Bocci et al., 2013; Lang et al., 2013; Rowinsky et al., 1989). Among these adverse events, hypersensitivity reactions were caused by the action of Cremophor^®^ EL (BASF Corp., Ludwigshafen, Germany) added to the treatment regimen to solubilise paclitaxel (Nehate et al., 2014; Picard & Castells, 2015).

The use of paclitaxel has not been frequently described in veterinary medicine owing to the high prevalence of acute hypersensitivity reactions to conventional Cremophor^®^ EL‐added drugs. In a study of dogs treated for cancer with paclitaxel at a dose of 165 mg/m^2^ through slow IV infusion every 3 weeks, hypersensitivity reactions were frequent (64%) despite pre‐treatment with corticosteroids (Poirier et al., 2004). Although a low dose of 132 mg/m^2^ has been suggested for minimising adverse events (Poirier et al., 2004), the high prevalence of hypersensitivity reactions has made paclitaxel with conventional Cremophor^®^ EL administration to veterinary cancer patients challenging. To overcome this difficulty, substances that do not cause hypersensitivity reactions when added to paclitaxel have been developed for safe drug delivery. Novel paclitaxel formulations have been designed to increase water solubility and reduce the risk of hypersensitivity reactions using non‐Cremophor^®^ formulations (Khanna et al., 2015).

The newly developed injectable paclitaxel has the advantage of safe delivery to cancer patients without hypersensitivity reactions and has been recommended as a therapeutic agent for various cancers in human patients (Miele et al., 2009; Stinchcombe, 2007). In contrast to several clinical trials in human medicine investigating treatment with new formulations of paclitaxel (Miele et al., 2009; Stinchcombe, 2007), only a few studies exist in the field of veterinary medicine. A water‐soluble micellar paclitaxel (Paccal Vet) formulation showed an effect on canine haemangiosarcoma in vitro (Reckelhoff et al., 2019). Subcutaneous administration of non‐Cremophor^®^ paclitaxel to dogs diagnosed with cancers showed anti‐cancer effects similar to those of conventional paclitaxel (Taxol^®^) and a low prevalence of hypersensitivity reactions (Selting et al., 2018; Silva et al., 2015).

Among the newly developed formulations of paclitaxel, the paclitaxel developed for oral intake has the advantage of easy administration as well as a longer exposure in human patients with various cancers, which is an important factor in the efficacy of cell‐cycle phase‐specific agents such as paclitaxel (Kang et al., 2018; Ryu et al., 2017). In a previous study conducted in human patients with advanced gastric cancer, the efficacy of oral paclitaxel was similar to that of conventional chemotherapy drugs, with a notably low prevalence of hypersensitivity reactions (Kang et al., 2018).

Although oral administration of paclitaxel to dogs with bladder cancer in a previous study conducted in our lab suggested the possibility of safe administration with minimal adverse effects (Chae et al., 2020), no study exists on the long‐term efficacy and safety of oral paclitaxel treatment on dogs with various cancers. Therefore, we aimed to retrospectively evaluate the efficacy and safety of oral paclitaxel in dogs with various cancers.

## 2 MATERIALS AND METHODS

### 2.1 Patient selection

This study was designed to evaluate the efficacy and safety of oral paclitaxel (DHP107‐VP; licensed by the Korean Food and Drug Administration; Daehwa Pharmaceutical Company Co., Ltd., Seoul, Republic of Korea) in dogs with various cancers. We reviewed the electronic medical records of dogs who were administered paclitaxel between May 2017 and August 2021 at our institution. Information obtained from medical records included signalment, cancer type, duration and number of paclitaxel administrations, response to therapy, treatment toxicity and duration of survival. The duration of paclitaxel administration was defined from the date of the first prescription to the last date for which the adverse event of the drug could be assessed. Dogs who received concurrent cytotoxic chemotherapeutic agents or could not be evaluated for safety after administration were excluded from the study. Dogs co‐administered with non‐steroidal anti‐inflammatory drugs (NSAIDs) were included in the study. Cancers were diagnosed using biopsy or cytology. The disease was staged through a complete blood count (CBC), biochemistry profile, urinalysis, diagnostic imaging, such as radiographs (thoracic and abdominal), abdominal sonography and elective computed tomography.

### 2.2 Treatment protocol

**FIGURE 1:**
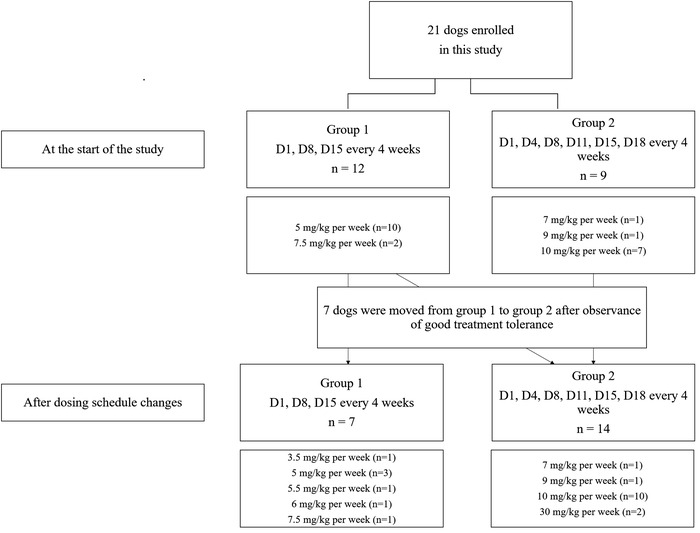
Flow diagram of changes in group composition of dogs enrolled in this study

Owing to the lack of studies on the protocol for oral paclitaxel dosing in veterinary medicine, the therapy was started with a low dose, which was adjusted according to each dog's response and condition. The dosing schedule was established by referring to the clinical trial results of oral paclitaxel in patients with various cancers in human medicine and various research studies on experimental animals (Chae et al., 2020; Hahn et al., 2014; Kang et al., 2018; Pallis et al., 2008). Dogs were administered oral paclitaxel three times a month (group 1) or six times a month (group 2). The dogs in group 1 received paclitaxel orally on days 1, 8, and 15 over 4 weeks. Dogs in group 2 were administered the drug on days 1, 4, 8, 11, 15, and 18 over 4 weeks. For a greater anti‐cancer effect, group movement was performed in some dogs (Figure 1).

During the treatment, drug‐related adverse events were evaluated by a veterinarian at each visit for the determination of the next dosing regimen. When no major side effects were observed at the initial dose, the dose was gradually increased until tolerated, as evaluated according to the absence of adverse events such as bone marrow suppression. The final dose was determined according to cancer progression and the patient's condition. The maximum weekly dose was set not to exceed 30 mg/kg, referring to the no‐observed‐adverse‐effect‐level of 5 mg/kg/day or less in the 6‐week repeated‐dose toxicity study in beagle dogs conducted by a pharmaceutical company (unpublished data). Treatment was continued until confirmation of significant disease progression, withdrawal of the owner's consent, or unacceptable toxicity, such as life‐threatening adverse events, were identified.

### 2.3 Efficacy and safety evaluation

To evaluate the efficacy of oral paclitaxel, imaging tests were performed for all dogs at intervals of 1–2 months throughout the drug delivery period. Treatment responses were classified as complete remission (CR, disappearance of all measurable lesions for > 4 weeks), partial remission (PR, >30% decrease in the sum of the largest target lesion diameters and no development of a new lesion for >4 weeks), stable disease (SD, neither sufficient shrinkage to qualify for PR or CR nor sufficient increase to qualify for PD for at least 4 weeks) or progressive disease (PD, >20% increase in the sum of the largest target lesion diameters or appearance of a new lesion) according to the Response Evaluation Criteria for Solid Tumors in dogs (RECIST v1.0) (Nguyen et al., 2015). Overall survival (OS), progressive‐free survival (PFS) and overall response rate (ORR) were also used for evaluating efficacy. OS was defined as the time from the initiation of treatment to the date of death or euthanasia owing to disease progression or the dog's last follow‐up. PFS was defined as the time from the initiation of treatment until disease progression. ORR was defined as the proportion of patients who had a complete or partial response to therapy. At the last data survey (August 2021), two dogs were alive and had not experienced progressive disease. These two cases were censored in the OS and PFS analyses. In addition, five cases in which cancer progression was not objectively imaged were also excluded from the investigation of PFS. Toxicity resulting from paclitaxel administration was evaluated retrospectively according to the CBC and patient history at each visit. Neutropenia and gastrointestinal adverse events were classified according to the criteria of VCOG‐CTCAE (LeBlanc et al., 2021). On long‐term administration of the drug >8 weeks in dogs, evaluation of liver/kidney dysfunction was conducted through regular blood analysis.

### 2.4 Statistical analysis

Statistical analyses were performed using Prism v.8.02 software (GraphPad Software Inc., La Jolla, CA, USA). The Shapiro–Wilk test was used to perform a normality test for age, OS and PFS. OS and PFS did not follow normal distribution and is expressed as a median value. Treatment responses, including CR, PR, SD, PD and ORR, were expressed by rounding to the second decimal place. Dogs that crossed over to group 2 during treatment were considered as part of group 2 in the final analysis. After performing a Kaplan–Meier analysis, the OS and PFS were compared between the groups using log‐rank tests. Fisher's exact test was used to compare the difference in the incidence of adverse events between the groups. Statistical significance was set at *p* < 0.05.

## 3 RESULTS

### 3.1 Patient characteristics

Twenty‐one dogs who received oral paclitaxel were included in the analysis. Of the 21 dogs, 2 were undergoing treatment at the time of the study, and the remaining died or discontinued follow‐up. Cancer diagnosis of enrolled dogs was made by cytology (*n* = 13) or biopsy (*n* = 5) or both (*n* = 3). In 16 of 21 dogs, oral paclitaxel treatment was started immediately without any other treatment within one month after diagnosis. The remaining five dogs were converted to oral paclitaxel after preceding treatment with surgery (*n* = 1), chemotherapy (*n* = 2; vinblastine for TCC treatment, both cases) or metronomic chemotherapy (*n* = 2; cyclophosphamide and toceranib phosphate, respectively). The dogs included 7 castrated males, 11 spayed females and 3 intact females. The patient's age ranged from 5 to 16 years (median: 12 years) and their weight ranged from 1.67 to 41.00 kg (median: 4.73 kg). The cancer types were transitional cell carcinoma (TCC, *n* = 5), oral squamous cell carcinoma (SCC, *n* = 2), pulmonary carcinoma (*n* = 3), mammary gland carcinoma (*n* = 3), renal cell carcinoma with pulmonary metastasis (*n* = 1), lymphoma with pulmonary involvement (*n* = 1), apocrine ductal carcinoma with pulmonary metastasis (*n* = 1), metastatic cancer of unknown primary origin (*n* = 1), intra‐abdominal malignant mesenchymal cell tumour (*n* = 1), inflammatory mammary gland carcinoma (*n* = 1), nasal carcinoma (*n* = 1) and nasal squamous cell carcinoma (*n* = 1). The breeds of dogs were Maltese (*n* = 9), Shih‐Tzu (*n* = 3), mixed dogs (*n* = 3), Yorkshire Terrier (*n* = 2), Cocker Spaniel (*n* = 2), Bichon Frise (*n* = 1) and Golden Retriever (*n* = 1).

### 3.2 Treatment results

Dogs received a median of 14 doses (range: 2–160 doses) of oral paclitaxel. The duration of administration was median 62 days (range: 8–813 days), and most showed a distribution proportional to the number of administrations. For dogs undergoing treatment at the time of investigation, the number of doses until August 2021 were included in the analysis. The number of doses that could not be used to determine the adverse events after prescription was excluded from the count. Overall, 625 doses of oral paclitaxel were administered. Based on the dosing schedule, the dogs were categorised into two groups: group 1 (paclitaxel administered on days 1, 8, and 15 every 4 weeks) and group 2 (paclitaxel administered on days 1, 4, 8, 11, 15, and 18 every 4 weeks). Initially, 12 dogs were in group 1 and 9 dogs were in group 2. After evaluating the occurrence of adverse events among the dogs in group 1, some dogs were moved to group 2 for the purpose of increasing anti‐cancer efficiency through dense dose delivery. In the final analysis, 7 dogs were in group 1, and 14 dogs were in group 2 (Figure 1). The median dose of oral paclitaxel delivered was 10 mg/kg per week (range: 3–30 mg/kg) in both groups.

The treatment was discontinued due to a progressive course of disease (*n* = 9), loss of follow‐up (*n* = 5), occurrence of neutropenia (*n* = 2) and death (*n* = 3) in 19 of 21 dogs at the time of investigation.

**FIGURE 2:**
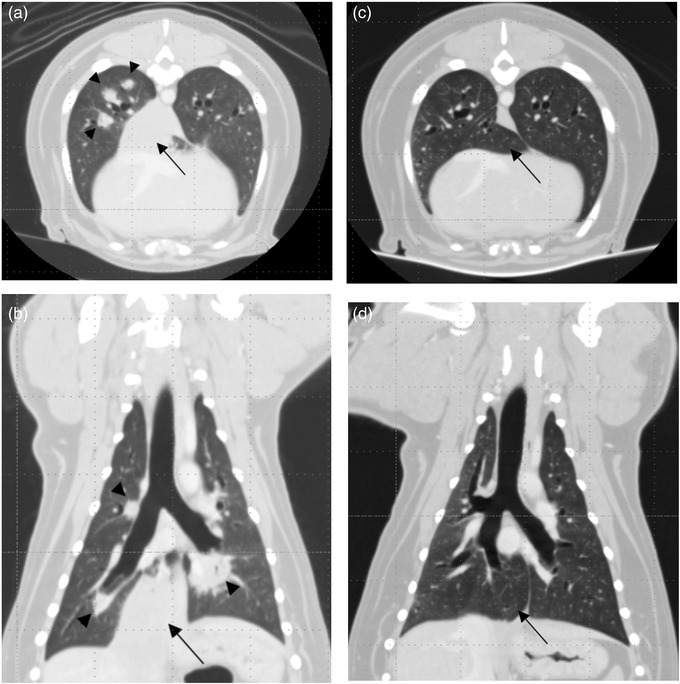
(a) Transverse and (b) dorsal plane computed tomography (CT) images of a dog with a relatively large mass of soft tissue that appeared as attenuation at the region of accessory lung lobe with a small, central air bronchogram (arrow) and multi‐focal small nodules of soft tissue attenuation (arrowheads). Primary lung tumour was diagnosed as pulmonary carcinoma by ultrasound‐guided fine needle aspiration. (c) Transverse and (d) dorsal plane CT images of the same dog after 10 months of oral paclitaxel administration. The previous attenuation at the accessory lung lobe was no longer visible (arrow) and small lung nodules in the lung field disappeared

Tumour size assessment following initiation of treatment in 16 dogs could be evaluated by RECIST v 1.1. The ORR was 6.25% in dogs capable of evaluating anti‐cancer response through RECIST. One dog achieved CR and 9 dogs (56.25%) achieved SD. The dog with pulmonary carcinoma achieved CR when confirmed by CT about 2 months after the initial administration of oral paclitaxel, and CR was maintained for more than 553 days until the time of investigation (Figure 2a–d). Nine dogs achieved SD, and their diagnoses were TCC (*n* = 4), oral SCC (*n* = 1), mammary gland carcinoma (*n* = 2), lymphoma with pulmonary involvement (*n* = 1) and pulmonary carcinoma (*n* = 1). The SD duration of dogs that achieved SD was median 148 days (range: 36–741 days). When oral paclitaxel was administered to dogs with TCC, the SD was 80%, the median OS was 284.5 days (range: 71–824 days) and the median PFS was 148 days (range: 22–741 days). The number of dogs co‐administered with non‐steroidal anti‐inflammatory drugs (NSAIDs) was five, all of which were diagnosed with TCC.

The median OS and PFS were 74 (range: 8–824 days) and 60.5 days (range: 14–741 days), respectively. The median OS and PFS in group 1 were 144 (range: 30–554 days) and 145 (range: 36–477 days), and those in group 2 were 88 (range: 8–824 days) and 25 (range: 14–741 days), respectively. When comparing the groups using the log‐rank test, OS and PFS did not reach statistical significance (*p* values were 0.9650 and 0.6543, respectively). The estimated hazard ratios for OS and PFS in group 1/group 2 were 0.9792 and 0.7818, respectively (95% confidence intervals: 0.3736–2.567 and 0.2584–2.365, respectively) (Table 1).

**TABLE 1:** Median overall survival (OS), progression‐free survival (PFS) according to group 1 and 2 and *p* value and hazard ratio between groups

Group	1	2	*p* Value	Hazard ratio (group 1/group 2)
Median OS	144 (*n* = 6)	88 (*n* = 13)	0.9650	0.9792
	(30–554 days)	(8–824 days)		
Median PFS	145 (*n* = 4)	25 (*n* = 10)	0.6543	0.7818
	(36–477 days)	(14–741 days)		

### 3.3 Adverse events

Most dogs tolerated oral paclitaxel well. The adverse events observed were bone marrow suppression (*n* = 3), vomiting (*n* = 2) and diarrhoea (*n* = 3) (Table 2). However, they were mostly mild (VCOG grade 1–2), and clinical signs resolved following drug dose reduction (*n* = 3), drug discontinuation (*n* = 3) and symptomatic treatment (*n* = 2). Neither the drug delay duration nor the symptomatic treatment duration was long (duration, <1 week). In the case of dose reduction, the dose was reduced to the dose prior to gradual escalation owing to the absence of a recommended dosing regimen and protocol in dogs. There was no significant difference in the occurrence of adverse events according to the paclitaxel administration group (*p* = 0.346).

**TABLE 2:** Dosage and grade of observed adverse events following oral paclitaxel administration

Dose		Neutropenia	Vomiting	Diarrhoea
6 mg/kg/week	Group 1		Grade 1 (*n* = 1) Recovery after dose reduction (5 mg/kg/week)	
7 mg/kg/week	Group 1	Grade 1 (*n* = 1) Recovery after 1 week of withdrawal. Restart with reduced dose		
	Group 2	Grade 1 (*n* = 1) Recovery after 1 week of drug discontinuation		
7.5 mg/kg/week	Group 1	Grade 2 (*n* = 1) Recovery after 1 week of drug discontinuation	Grade 1 (*n* = 1) Recovery after dose reduction (5 mg/kg/week)	
10 mg/kg/week	Group 2			Grade 1 (*n* = 1) Recovery after symptomatic treatment. Restart with same dose Grade 2 (*n* = 1) Recovery after symptomatic treatment. Restart with same dose
15 mg/kg/week	Group 2			Grade 1 (*n* = 1) Recovery after dose reduction

*Note*: Haematological and gastrointestinal toxicity was evaluated after oral paclitaxel dosage adjustment, adapted from the Veterinary Cooperative Oncology Group‐Common Terminology Criteria for Adverse Events

## 4 DISCUSSION

This study was intended to evaluate the safety and efficacy of oral paclitaxel in dogs with naturally occurring cancers. Although proving the efficacy of oral paclitaxel in this study was difficult, it was confirmed that it is a relatively safe drug without life‐threating hypersensitivity as that seen with conventional Taxol.

The conventional formulation of paclitaxel is an IV injection product solubilised in Cremophor EL^®^ and ethanol (Nehate et al., 2014; Picard & Castells, 2015). Cremophor EL^®^ and ethanol can solubilise the poorly soluble paclitaxel and effectively deliver the drug to cancer cells to achieve anti‐cancer effects. However, they can cause strong hypersensitivity reactions due to their toxicity. Therefore, conventional paclitaxel requires pre‐treatment with steroids and anti‐histamines, and should be slowly infused for more than 3 h with serial monitoring. These drawbacks limited the use of conventional paclitaxel in dogs and cats with cancers (Kim et al., 2015; Poirier et al., 2004). Recently, a new paclitaxel formulation was developed for safe and efficient drug delivery using a non‐Cremophor EL^®^ formulation (Khanna et al., 2015; Nehate et al., 2014). The oral paclitaxel used in our study was solubilised using a mixture of monoolein, tricaprylin and polysorbate 80, which had a better safety profile compared to conventional formulation (Jang et al., 2017). The mucosal adhesive properties of this new formulation improve the permeability and contribute to the effective distribution of the drug in major organs after absorption in the gastrointestinal tract (Hong et al., 2007; Jang et al., 2017).

In terms of efficacy, it was difficult to demonstrate the efficacy of oral paclitaxel with statistical significance due to the lack of enrolled dogs and the characteristics of the retrospective study. Nevertheless, the results of our study suggest the potential use of oral paclitaxel in the treatment of dogs with TCC and pulmonary carcinoma. Determining the clear effect of paclitaxel in TCC was challenging due to the small number of cases and co‐administration with NSAIDs; however, our findings are similar to those reported in a previous oral paclitaxel case report (Chae et al., 2020) that suggested the possibility of using oral paclitaxel as a treatment for dogs with TCC. In addition, in dogs with pulmonary carcinoma, CR was observed in one case and SD was noted for more than 497 days in another case. Although further studies are needed, these two cases support the possibility of effective and safe administration of oral paclitaxel for dogs with pulmonary carcinoma, especially since paclitaxel has been used for non‐small lung cancer in humans (Akerley III, 2000; Hajek et al., 1996). However, many dogs enrolled in this study exhibited PD despite oral paclitaxel administration. This low treatment response may be attributed to lack of a therapeutic effect of this drug on the other types of cancers; however, it could also be associated with the difficulty in distinguishing the treatment effect because some dogs already had cancers in a terminal state. In fact, 6 of the enrolled dogs were treated with oral paclitaxel in an already metastatic state. Oral paclitaxel was preferred in these metastasised dogs owing to convenient dosing and fewer hospital visits; however, it is thought that this study may have resulted in a low response rate. In addition, this low response rate could be attributed to the inclusion of non‐epithelial‐derived cancer cases in this study, although taxanes are proven therapeutic agents chiefly for epithelial‐derived cancer in human medicine (Maloney et al., 2020).

For the safety evaluation, adverse events were graded retrospectively and oral paclitaxel was noted to be a safe drug with only minor adverse events. Gradual dose changes were performed in this study owing to the lack of an established safe therapeutic dose for maximal efficacy in dogs with naturally occurring cancers. The observed adverse events were gastrointestinal symptoms and bone marrow suppression. Of the three cases of bone marrow suppression, two cases were VCOG grade 1 and 1 case was VCOG grade 2. Of the five cases of gastrointestinal symptoms, only one case of diarrhoea was VCOG grade 2, and all the others had mild symptoms of VCOG grade 1. All symptoms resolved after temporary drug discontinuation, dose reduction or symptomatic treatment such as anti‐diarrheal drugs, and fluid therapy. In addition, no liver or kidney toxicity associated with drug accumulation was demonstrated in dogs administered long‐term paclitaxel for 8 weeks or more. In contrast to the high rate of hypersensitivity reactions that occurred when conventional Taxol was administered to dogs (Kim et al., 2015; Poirier et al., 2004), no hypersensitivity reactions were observed with oral paclitaxel. The safety of oral paclitaxel administration without pre‐treatment signifies that it can be considered as a treatment for dogs diagnosed with various cancers.

In addition to demonstrating the safety of the drug and suggesting its potential as a therapeutic agent in certain cancers, this study presents an appropriate drug‐delivery protocol for dogs diagnosed with cancer owing to the lack of well‐established protocols in small animals. A delivery schedule of days 1, 8 and 15 every 4 weeks was applied to patients in group 1 based on the results of a previous study by Kang et al. (2018). The protocol in group 2 was established with reference to previous studies that showed superior effects with metronomic drug delivery at low doses twice a week than drug delivery at high doses once a week in an orthotopic mouse model of ovarian cancer (Hahn et al. 2014). There was no difference in the prevalence of adverse events between treatment schedules of three times a month or six times a month in this study. Considering the drug characteristic of cell‐cycle specific (Kang et al. 2018) and the results of adverse events occurrence between the two groups, twice a week dosing is appropriate for dense dose delivery. Further studies should be conducted to establish a safe dose with the maximum effect. If an appropriate therapeutic dose and protocol with the maximum anti‐cancer effect are established through a larger‐scale study, faster therapeutic effects and better outcomes can be expected.

This study had some limitations. First, a small number of dogs were investigated for each cancer, making this study inadequate to definitively prove the efficacy of oral paclitaxel in dogs with various cancers. Further studies involving more dogs diagnosed with cancer are needed. Second, this study was conducted retrospectively, making it difficult to investigate the therapeutic effect under the same conditions. Important constitutional and gastrointestinal events that occurred between visits may not have been well captured in the medical records through retrospective evaluation. Thus, further prospective studies at specific cancer types and stages are needed. Third, since there was no control group in this study, it was difficult to distinguish the therapeutic effect in the treatment groups. Finally, the drug doses and treatment schedules were different among the subjects. Considering that a sub‐therapeutic dose may have been administered to some patients, it was difficult to consistently evaluate adverse events and efficacy due to dose variability. Although this study suggests a gradual increase in the dose through twice‐weekly administration, further studies are needed to determine a safe drug setting for maximum effect.

## 5 CONCLUSION

Although the efficacy of the drug in various cancers has not been demonstrated, this study supports the possibility that oral paclitaxel could be used as an effective drug for TCC and pulmonary carcinoma. In addition, it was safely administered with minimal toxicity to dogs compared to IV injection. Further studies on specific cancers and stages in companion animals should be conducted to demonstrate the potential of oral paclitaxel in the effective treatment of animals diagnosed with various cancers.

## ETHICS STATEMENT

The authors confirm that the ethical policies of the journal, as noted on the journal's author guidelines page have been adhered to.

## CONFLICT OF INTEREST

The authors declare that they have no competing interests.

## AUTHOR CONTRIBUTIONS

HK contributed to conceptualisation, investigation, resources, writing‐original draft; SM and JH contributed to writing‐review and editing; KY contributed to radiology advice and writing‐reviews; SN contributed to the provision of drug information, resources; YI, KW and HW contributed to supervised the procedures. All the authors have read and agreed to the published version of the manuscript.

### PEER REVIEW

The peer review history for this article is available at https://publons.com/publon/10.1002/vms3.829.

## Data Availability

The data that support the findings of this study are available from the corresponding author upon reasonable request.
